# Mother's perception regarding insufficiency of breast-milk: a qualitative investigation in Haripur, Pakistan

**DOI:** 10.3389/fpubh.2026.1742704

**Published:** 2026-03-24

**Authors:** Ijaz ul Haq, Waqar Khan, Cui Hou, Hasnat Nisar, Shahbaz Ahmad Zakki, Madiha Bibi, Xiaoyun Wang, Xiaojing Hu

**Affiliations:** 1 Fujian Key Laboratory of Neonatal Diseases, Xiamen, Fujian, China; 2 Nursing Department, Children's Hospital of Fudan University, Shanghai, China; 3 Department of Public Health & Nutrition, The University of Haripur, Haripur, Khyber Pakhtunkhwa, Pakistan; 4 Department of Clinical Nutrition, College of Applied Medical Sciences, King Faisal University, Al-Ahsa, Saudi Arabia; 5 Inner Mongolia Maternal and Child Health Care Hospital, Hohhot, China; 6 School of Health Sciences Peshawar, Khyber Medical University, Peshawar, Pakistan

**Keywords:** breast milk, challenges, insufficiency, perception, qualitative study

## Abstract

**Background:**

Breast milk serves as the primary source of nutrition for infants, playing a crucial role in enhancing their immunity against viruses and bacteria. Insufficient breast milk may lead to impaired growth in babies. The resent study aims to explore mothers' perceptions and challenges associated with breast milk insufficiency in Haripur District, Pakistan.

**Methods:**

In this qualitative investigation, 60 mothers from diverse hospitals in the Haripur district were interviewed via a semi - structured interview protocol. The responses were documented through audio recording and note - taking. Thematic analysis was employed to examine the interview data with the utilization of N-Vivo 11.0 software.

**Result:**

Mother's perception regarding breastmilk insufficiency revealed three primary themes: Factors Influencing Breastfeeding Practices, Breastfeeding Outcomes and Challenges, and Breastfeeding Advice and Support. Multiple factors, such as cesarean section, health issues, skin - to - skin contact, insufficient support, and knowledge deficiency, were recognized as influencing breastfeeding practices and outcomes. A significant number of mothers reported encountering challenges in exclusive breastfeeding and initiating breast milk production. Respondents had diverse experiences regarding family support, with the majority of families being supportive while some were not. The majority of respondents expressed satisfaction with hospital services.

**Conclusion:**

This study provides valuable insights into multiple factors that must be considered to prevent breast milk insufficiency. These factors include delayed onset of breastfeeding, a lack of breastfeeding knowledge, inadequate family support, health conditions, and delivery modes such as cesarean section. When formulating strategies, policymakers should take these challenges into consideration.

## Introduction

Breastfeeding is considered the primary source of nutrition, offering numerous health benefits and providing the most appropriate nutrients for growth and development (1). Breastfeeding reduces postpartum depression, decreases maternal stress, and improves the physical health of both the mother and infant (2). According to WHO guidelines, newborns should be exclusively breastfed for the first six months, followed by complementary feeding until the age of two years (3). In both high-income and low-income countries, breastfeeding reduces the risk of obesity and chronic diseases later in life while supporting healthy brain development. It is also crucial for preventing the triple burden of infectious diseases, mortality, and malnutrition (4).

Breast milk reduces the occurrence of chronic illnesses, including allergies, asthma, and diabetes, while protecting infants against infectious diseases such as diarrhea, respiratory infections, ear infections, and urinary tract infections during their first year of life (4). Additionally, breastfeeding practices serve as a preventive measure against ovarian and breast cancers (5, 6).

According to the Pakistan Demographic and Health Survey (PDHS), only 40% of children under six months of age are exclusively breastfed, and 53% of children continue breastfeeding until the age of two (7). WHO aims to increase the rate of exclusive breastfeeding during the first six months to at least 50% by 2025, up from the current rate of 40% (8).

Despite its significance, breastfeeding presents numerous challenges for mothers. The most prevalent among these are the perception of insufficient milk production and the apprehension that they might not generate an adequate quantity of milk to fulfill their infants‘ requirements (9). This conviction may lead to subsequent inadequate breastfeeding and is often correlated with infant malnutrition (10). Mothers' perceptions of breast milk insufficiency are shaped by various factors, including community culture, family support, hospital practices, and maternal psychological wellbeing (11). Consequently, formula feeding and the early discontinuation of breastfeeding are frequently driven by the perceived insufficiency of milk supply (8).

Additional barriers to sustained breastfeeding include full-time employment, widespread misconceptions about lactation, concerns regarding maternal-infant bonding interference, and perceived insufficient milk supply (12). Exclusive breastfeeding is affected by a diverse range of sociodemographic, clinical, and psychosocial factors, such as insufficient awareness, heavy occupational workload, low socioeconomic status, mode of childbirth, infant gender, absence of partner support, and physiological lactation difficulties (10). Furthermore, maternal dietary behaviors and eating disorders have been shown to adversely impact breastfeeding initiation and duration (13).

The most commonly reported breastfeeding challenges encompass nursing-related pain, nipple trauma, and the perception of insufficient milk production. Evidence indicates that coordinated, multidisciplinary care significantly improves breastfeeding outcomes and enhances maternal satisfaction (14). Notably, breastfeeding rates exhibit a substantial increase when mothers are provided with comprehensive, evidence-based support, which includes early discharge planning, oral stimulation techniques, systematic breastfeeding education, in-hospital lactation assistance, early initiation of breastfeeding, kangaroo mother care, and the institutional implementation of supportive policies and educational programs (6).

Pakistan, as a resource-limited setting, faces substantial challenges related to perceived and actual breast milk insufficiency, which are associated with serious adverse outcomes including childhood morbidity, mortality, and both acute and chronic malnutrition. Addressing this issue is critical for improving maternal and child health indicators. Particularly in Khyber Pakhtunkhwa, there remains a pressing need for context-specific research to identify the multifactorial barriers contributing to suboptimal breastfeeding practices. A clear understanding of these barriers is essential for designing evidence-based interventions to prevent early weaning and mitigate long-term nutritional deficits. This qualitative study investigates the determinants of breast milk insufficiency among mothers in Haripur District, Khyber Pakhtunkhwa, Pakistan. Haripur, situated at the intersection of Khyber Pakhtunkhwa, Punjab, and near Islamabad, hosts a linguistically and culturally diverse population, including speakers of Urdu, Punjabi, Hindko, and Pashto. Its proximity to major urban centers results in frequent healthcare utilization across multiple hospitals. By exploring maternal experiences and systemic challenges in this under-researched region, the study aims to generate actionable insights. The findings will inform targeted public health strategies to strengthen breastfeeding support systems and reduce malnutrition rates in Haripur and comparable low-resource settings.

## Materials and methods

### Study design

The current study was a qualitative exploratory study that utilized a semi - structured interview approach to investigate mothers' cognitions regarding postpartum breastfeeding insufficiency. Spanning from January to March 2024, the study was executed in multiple hospitals within the Haripur district, including DHQ Hospital Haripur, Yahya Hospital, Mother Care Hospital, Urban Dispensary Haripur, Mehr General Hospital, and Allama Iqbal Hospital Haripur. A purposive sampling strategy was used to select participants whose infants were six months of age or younger.

### Participants

The source population was defined as mothers of infants who were admitted to the aforementioned public and private hospitals in Haripur. The study subjects were individual mothers, with whom in - depth interviews were conducted regarding breast milk insufficiency. Participants were recruited from the gynecological wards of various hospitals in Haripur district, Khyber Pakhtunkhwa, including both mothers who had vaginal deliveries and those who underwent Cesarean sections. The majority of participants were residents of the Haripur district.

### Inclusion and exclusion criteria

The research included mothers with infants aged up to six months. Eligible participants were those willing to volunteer and able to provide informed consent. Exclusion criteria comprised mothers with cognitive impairments, language barriers, or communication difficulties.

### Data collection

Data for this study were collected through semi-structured interviews with 60 mothers recruited from multiple hospitals in Haripur District. Interview followed the stauration, Interviews followed an open-ended questionnaire outlined in the interview guide (see Table 1) and were audio-recorded to ensure data accuracy and completeness. Each interview endured for a duration ranging from 30 to 45 min, offering profound understandings of the participants' experiences and viewpoints.

**Table 1:** Interview guide.

**Good day. I sincerely appreciate your participation in this interview. During this interview, I aim to gain a more in - depth understanding of your breastfeeding experiences subsequent to childbirth. Is that acceptable?**
• Could you please describe your breastfeeding experience and specify the feeding method you selected for your newborn infant? • How was skin-to-skin contact conducted before the first breastfeeding following childbirth? When did breastfeeding initiation occur after birth, and what were your experiences and feelings during this process? • Could you describe your perception of whether your breast milk supply was sufficient to meet your infant's needs? • Could you describe your experience with practicing exclusive breastfeeding, defined as providing only breast milk without any additional fluids or supplements? • Could you describe whether you received any advice, education, or support from healthcare providers, family members, or friends regarding concerns about insufficient breast milk, and how you experienced or made sense of this support? • Could you share how perceptions about milk supply adequacy influenced your breastfeeding decisions and practices? • What personal, social, or environmental circumstances during breastfeeding felt particularly challenging, and how did you emotionally navigate those experiences? • How do you characterize your confidence in fulfilling maternal responsibilities? What reflections or emotions emerge when contemplating your parenting role? • How satisfied are you with the breastfeeding-supportive facilities available in the hospital setting? • Which standard hospital routines during your postpartum stay created unexpected difficulties for breastfeeding initiation or maintenance? • What self-care rituals, thought patterns, or relational interactions helped you preserve emotional equilibrium during breastfeeding challenges? • Could you walk me through your nutritional habits and hydration practices while lactating? • What coping strategies or personal practices helped reduce your stress levels and enhance relaxation during this period?

During each interview, responses were systematically recorded, verbatim transcribed, and thematically summarized to capture key insights. This methodological approach enabled a comprehensive exploration of maternal experiences and perceptions related to perceived or actual breast milk insufficiency in the postpartum period.

### Interview guide

The interview guide for this study was developed by experienced researchers, focusing on open-ended questions addressing breast milk insufficiency, exclusive breastfeeding, maternal nutrition, infant feeding practices, the breastfeeding environment, and family support. To ensure content relevance and clarity, the research team reviewed existing literature to identify key knowledge gaps and conducted a pilot interview with five mothers, which informed iterative refinements to the guide; data from these pilot interviews were excluded from the final analysis.

Participants were informed about the nature and purpose of the questions prior to participation, and interviews were conducted in the participants' preferred languages — Urdu, Hindko, or Pashto—to enhance comprehension and comfort. Initial questions collected demographic information, including maternal age, infant age, occupation, education level, infants birthplace, and parity. The subsequent semi-structured questions are detailed in Table 1 of the interview guide.

### Data analysis

Thematic analysis was utilized to scrutinize the interview data through the use of NVivo 11.0 software. The audio - recorded files and interview documents were methodically arranged and compiled via Microsoft Word and subsequently transcribed word - for - word. Researchers initiated the process by comprehensively immersing themselves in the data through multiple readings of interview transcripts and making preliminary annotations to understand the overall context, record initial impressions, and avoid preconceived assumptions. Subsequently, each sentence or segment was openly coded using NVivo to generate initial codes. Related codes were grouped into candidate themes, which were refined by comparing them with the original data to ensure accuracy. Irrelevant or redundant themes were removed, logical connections between themes were identified, and a hierarchy of core and sub - themes was established to reflect the depth of the findings.

## Results

**Figure 1:**
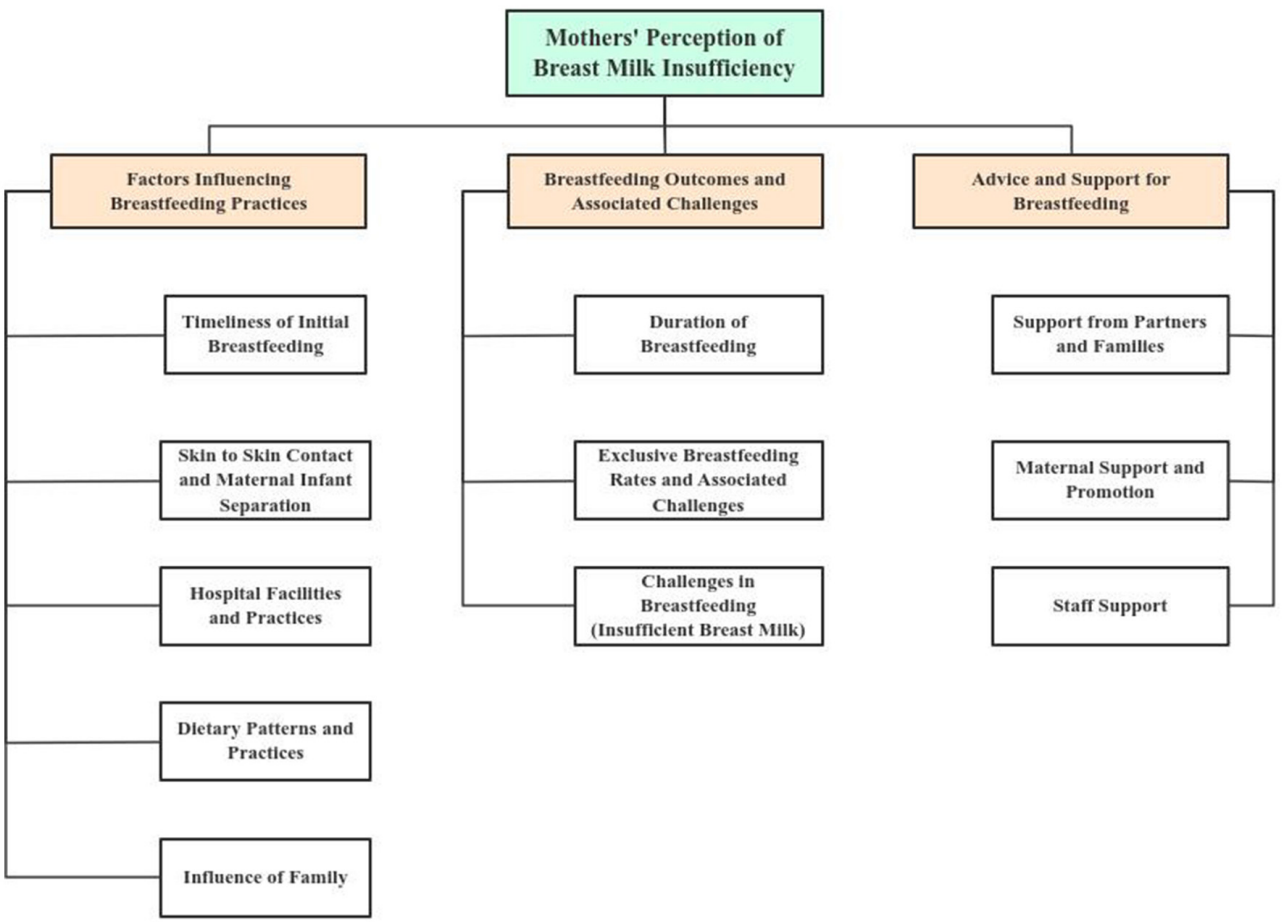
Main themes and their sub – themes.

The demographic characteristics of the population are presented in Table 2. The majority of the respondents (50.0%) fell within the 26–35 age group. Sixty - three percent had infants under 2 months of age, 26.7% were illiterate, and 95% were residents within the same district. In the present study, 60 individuals were subsequently interviewed (Supplementary File, S1). Mothers' perceptions regarding breastmilk insufficiency revealed three primary themes: Factors Influencing Breastfeeding Practices, Breastfeeding Outcomes and Challenges, and Breastfeeding Advice and Support. These primary themes were classified into various related sub - themes, as depicted in Figure 1.

**Table 2:** Demographic characteristics of the respondents.

**Variables**	**Frequency**	**Percentage**
**Age of mothers (years)**		
18–25	26	43.3
26–35	30	50.0
36–40	4	6.7
**Age of children (months)**		
0–2	38	63.3
3–4	15	25.0
5–6	7	11.7
**Educational status**		
Primary	22	36.7
Secondary	7	11.7
Tertiary or more	15	25.0
No-education	16	26.7
**Occupation**		
Housewife	60	100.0
**Number of children**		
1–5	58	96.7
6–10	2	3.3
**Delivery places**		
Haripur	51	85.0
DHQ	9	15.0
**Districts**		
Haripur	57	95.0
DHQ	3	5.0

### Factors influencing breastfeeding practices

#### Timeliness of initial breastfeeding

One of the significant factors affecting breastfeeding behaviors is the initiation timing. Specifically, merely 20% of mothers (*n* = 12) initiated breastfeeding for their infants within the first hour after birth, whereas 80% (*n* = 48) failed to commence breastfeeding within this crucial time period.

“*My baby was a little weak at first, so we got separated and I couldn't breastfeed right away” (R16)*.

A significant number of these respondents indicated that they had undergone cesarean section, leading to the non - onset of lactation at that time.

“*No, I didn't breastfeed at first because I had a C-section — I started with bottle-feeding*.” *(R1)*.

#### Skin to skin contact and maternal infant separation

Approximately 61.67% of the mothers (*n* = 37) reported that they did not engage in skin - to - skin contact with their infants until after the first breastfeeding session. This finding implies that this practice was not widely adopted by the mothers.

“*No, I didn't get to have skin-to-skin with my baby until we actually started breastfeeding” (R12)*.

Approximately 18.33% (*n* = 11) of mothers engaged in skin - to - skin contact with their infants prior to breastfeeding, suggesting that a notable minority adhered to this practice.

“*Yeah, I got to have skin-to-skin with my baby right after birth” (R15)*.

Furthermore, approximately 20% of the respondents (*n* = 12) indicated that they did not engage in skin - to - skin contact because of undergoing a cesarean section.

“*No, I didn't do it because I had a C-section.” (R6)*.

This finding implies that this procedure was not typically adhered to by the participants.

#### Hospital facilities and practices

A high satisfaction rate of 95% was documented, wherein the majority of respondents (57 out of 60) indicated satisfaction with the support provided by the staff.

“*Everything went smoothly at the hospital — I was really happy with the staff, and they had all kinds of helpful facilities” (R11)*.

Nevertheless, three respondents (5%) voiced apprehensions, including problems related to the hospital's sanitation, the absence of an English-style commode, and unhygienic washrooms.

“*The staff gave me everything I needed, which was great, but I was a bit unhappy with how clean the hospital ward was” (R10)*.

“*The hospital had good facilities overall, but the bathroom didn't have a Western-style toilet, which was kind of inconvenient for me, and it wasn't very clean either” (R19)*.

The vast majority of respondents (*n* = 59, 98.33%,) indicated that routine hospital practices did not impede mothers engaged in breastfeeding. Nevertheless, a single respondent (1.66%, *n* = 1) reported experiencing interference, attributing it to the substandard conduct of hospital staff and nurses.

“*The nurses and hospital staff weren't very supportive, and it actually made it harder for me to breastfeed” (R18)*.

#### Dietary patterns and practices

During the lactation period, the majority of mothers typically consumed foods including dried fruits (*n* = 39, 65%), milk (*n* = 50, 83%), broth (*n* = 37, 66.6%), chicken (*n* = 30, 50%), and eggs (*n* = 16, 26.6%).

“*While I was breastfeeding, I mostly ate milk, chicken, and yakhni.” (R7)*.

Beverages, including tea (*n* = 22, 36.6%), juice (*n* = 10, 16.6%), and green tea (*n* = 8, 13.3%), were commonly consumed. Meanwhile, other food and drink items such as cake (*n* = 12, 20%), halwa (*n* = 8, 13.3%), bread (*n* = 18, 30%), fruits (*n* = 18, 30%), nuts (*n* = 8, 13.3%), vegetables (*n* = 10, 16.6%), biscuits (*n* = 4, 6.6%), paratha (*n* = 4, 6.6%), desi ghee (*n* = 6, 10%), kahwa (*n* = 10, 16.6%), soap (*n* = 6, 10%), Zam Zam water (*n* = 2, 3.3%), nashta (breakfast; *n* = 2, 3.3%), and dania (*n* = 2, 3.3%) were also consumed by a portion of mothers.

One respondent mentioned: “*While I was breastfeeding, I usually had milk, cake, yakhni, qehwa, and tea with bread*” *(R6)*.

Another respondent said: “*While I was breastfeeding, I usually ate meat, desi ghee, milk, eggs, dry fruits, chicken, and even soap*” *(R17)*.

The majority of mothers (*n* = 31, 51%) reported that they adhered to their regular home - based diet. They did not receive any additional or nutritionally enhanced meals from their families.

“*I just eat whatever's available at home” (R15)*.

#### Influence of family

A high proportion of 98.33% of mothers (*n* = 59) reported satisfaction with the support they received from their families during the breastfeeding phase.

One respondent mentioned: “*My family recommended breastfeeding for my baby — everyone was supportive, and it didn't affect my decision to go ahead with it*” *(R2)*.

Only a single mother (*n* = 1) voiced dissatisfaction regarding family support during the breastfeeding period owing to a familial conflict with her mother - in - law, which had an impact on her breastfeeding behaviors.

She stated: “*Yeah, I was dealing with some family issues — my mother-in-law wasn't really supportive, and that did end up affecting my decision to breastfeed” (R41)*.

### Breastfeeding outcomes and associated challenges

#### Duration of breastfeeding

Among the 60 mothers, 47 (78.33%) practiced exclusive breastfeeding for their infants.

One respondent mentioned: “*For six months, I fed my baby my own milk, and I also gave him cow's milk” (R20)*.

Another respondent said: “*For two days straight, I gave my baby only my own milk” (R21)*.

However, 13 mothers (21.67%) did not undertake any form of breastfeeding for their infants.

One mother explained: “*I don't give my baby my breast milk because I just don't have enough — I ended up going with bottle feeding instead”(R12)*.

Another respondent shared: “*I haven't breastfed my baby yet because I had a C-section — it kind of made it hard to get started”(R36)*.

A third mother said: “*I didn't breastfeed my baby because I'd had surgery and it was really hard to sit up at the time” (R55)*.

The findings indicated that the majority of the participants breastfed their infants, whereas a minor proportion did not.

#### Exclusive breastfeeding rates and associated challenges

In relation to breastfeeding rates and challenges, it is evident that the majority of respondents (*n* = 39, 65.00%) engaged in exclusive breastfeeding for their infants without any alternative feeding methods, which demonstrates a favorable tendency toward breastfeeding. Nevertheless, challenges were identified, as 21.67% of respondents (*n* = 13) did not breastfeed their infants at all and solely resorted to bottle - feeding, implying difficulties in sustaining exclusive breastfeeding. Moreover, 13.33% of respondents (*n* = 8) employed both breastfeeding and bottle - feeding. These findings underscore the necessity for additional support or resources to facilitate the overcoming of breastfeeding challenges.

“*I don't use any alternate for breast milk; I only use my own breast milk to feed my baby” (R1)*.

“*Sometimes, I breastfeed my baby, but at other times, I use bottle feeding” (R38)*.

“*I fed my baby with formula in a bottle; I didn't add anything else” (R12)*.

#### Challenges in breastfeeding (insufficient breast milk)

The majority of mothers (70.00%, *n* = 42) did not encounter difficulties during breastfeeding and reported a positive breastfeeding experience. Nevertheless, 30.00% (*n* = 18) of the mothers faced challenges. These difficulties were diverse, encompassing physical discomfort resulting from prior surgeries or bodily pain, such as that associated with a cesarean section. Some mothers voiced concerns regarding insufficient breast milk, which gave rise to anxiety and stress about their infants' nutrition. Others confronted health issues with their babies, such as jaundice or pneumonia, which impaired their ability to breastfeed effectively. Additionally, some respondents experienced household stressors or lacked family support, which further complicated the breastfeeding process.

“*Yeah, I've had a really tough time. I don't produce enough breast milk, and we can't afford formula for my baby. On top of that, there's just so much housework to get done” (R42)*.

“*Yeah, I've been having some problems — I had surgery, and sometimes I still feel pain in my body” (R49)*.

“*Yeah, it was hard for me at first because it's my first baby, and no one showed me how to hold or take care of him” (R54)*.

“*Yeah, I went through a lot. My baby had pneumonia, which made breastfeeding really difficult” (R55)*.

Nearly half of the respondents (*n* = 29, 48.33%) reported that they did not encounter any insufficiency in breast milk, whereas 51.67% (*n* = 31) experienced breast milk insufficiency. Multiple factors contributed to the reduction in milk production, encompassing cesarean section delivery, physical weakness, a dearth of knowledge and experience regarding dietary patterns, low milk yield, and non - feeding of the infant. Additionally, other factors such as maternal health issues, ineffective breastfeeding techniques, and cultural or social elements also had an impact on milk production.

“*Yeah, I feel like I don't have enough milk. After my C-section, I was really weak and didn't have much of an appetite because of my diet, so my supply stayed low” (R2)*.

“*I sometimes feel like I'm not making enough breast milk, especially when my baby doesn't latch or seems unsatisfied” (R8)*.

“*I used to cry a lot when I wasn't producing enough milk. I had a C-section and was in the hospital, so I felt really bad for my baby. I was super nervous that my milk wouldn't be enough” (R55)*.

### Advice and support for breastfeeding

#### Support from partners and families

Sixty-five percent of mothers (*n* = 39) reported receiving support from family members or friends during breastfeeding. This support included emotional encouragement, practical help with caregiving responsibilities, guidance on breastfeeding techniques, financial support, and motivation to adhere to a nutritious diet to promote adequate milk production.

“*Yeah, my family really supported me throughout my pregnancy — they even helped me financially” (R12)*.

“*Yeah, my mom, my mother-in-law, and my husband were all there for me — they took care of me a lot” (R54)*.

Nevertheless, 35% of mothers (*n* = 21) reported the absence of any support from family members or friends.

“*No, I haven't gotten any support from my family or friends” (R10)*.

“*No, there's nobody in my family who can help out with anything — I live alone, so I have to do everything on my own” (R35)*.

The absence of support can exert a detrimental influence on the breastfeeding experience and the comprehensive well - being of mothers. In the absence of sufficient assistance, mothers may encounter challenges in handling childcare duties and preserving their health during the breastfeeding phase, thereby giving rise to additional obstacles.

#### Maternal support and promotion

A substantial proportion of mothers, specifically 90% (*n* = 54), reported experiencing sentiments of happiness and satisfaction in relation to motherhood, which indicates their sense of fulfillment and contentment with maternal duties.

“*As a mom, I feel really great — my whole life has changed since my baby was born” (R34)*.

“*As a mom, I've been so much happier. My husband and I weren't getting along well before the baby came, but after he arrived, things between us got a lot better” (R43)*.

Nevertheless, 5% of mothers (*n* = 3) reported experiencing negative emotions or feelings of anxiety that affected their psychological wellbeing and self-esteem. These mothers highlighted challenges such as the burden of household responsibilities, insufficient breast milk supply, or infant feeding refusal.

“*As a mom, I do get a bit worried when my baby doesn't want to feed” (R17)*.

“*As a mom, I feel really tired — I've got housework to do and I'm the only one taking care of the baby” (R27)*.

“*Being a mother is such a hard job” (R47)*.

Additionally, another 5% of mothers (*n* = 3) reported feeling happy while simultaneously acknowledging specific challenges. These findings indicate that although most mothers derive joy from motherhood, they also actively recognize and cope with the associated difficulties. Overall, the data reveal a diverse spectrum of emotional experiences, underscoring the importance of accessible support systems and resources to assist mothers in navigating both the positive and challenging aspects of parenting.

#### Staff support

Of the 60 respondents, 59 (98.30%) reported satisfaction and provided positive feedback regarding the support they received from healthcare staff. This high level of satisfaction indicates that hospital personnel effectively met the majority of participants' support needs during their care experience.

“*I'm really happy with the staff — they helped me so much with breastfeeding and were super easy to talk to” (R32)*.

“*I was really happy with the hospital staff; they were great and gave me a lot of support with breastfeeding” (R57)*.

Nevertheless, 1.66% of mothers (*n* = 1) reported dissatisfaction, citing staff misconduct related to breastfeeding support and a lack of collaborative care as the primary reasons.

“*I wasn't happy with the staff because of how they acted — they weren't supportive and didn't really help me with breastfeeding” (R23)*.

## Discussion

This study explored maternal perceptions of breast milk insufficiency in Haripur, Pakistan. Nearly half of the mothers (51.67%, *n* = 31 out of 60) reported experiencing insufficient breast milk, highlighting a range of contributing factors to reduced lactation. These findings underscore the importance of addressing modifiable barriers and providing targeted support to improve breastfeeding outcomes postpartum.

Breast milk insufficiency significantly affects mothers' emotional, psychological, social, and physical wellbeing. In this sample, a substantial proportion of mothers experienced challenges with milk supply following childbirth. Key determinants included cesarean delivery, which delayed early breastfeeding initiation, as well as maternal physical weakness, limited knowledge about breastfeeding practices, prior negative breastfeeding experiences, and underlying health conditions. Additional influencing factors encompassed suboptimal dietary habits, absence of immediate skin-to-skin contact after birth, elevated levels of anxiety and stress, and household responsibilities or lack of familial support.

Breastfeeding is critical for infant health and development. Multiple factors can hinder successful breastfeeding in the immediate postnatal period. Previous research has identified lack of awareness and inadequate maternal knowledge regarding optimal breastfeeding techniques as primary barriers to exclusive breastfeeding (15), findings that align closely with those of the present study. Breast milk serves as the primary source of essential nutrients for newborns; insufficient intake due to low milk supply may contribute to chronic malnutrition, including stunting (16). Addressing lactation challenges requires improving nutrition literacy, promoting adherence to early breastfeeding guidelines, and removing structural and sociocultural obstacles to optimal feeding practices. Prior studies have documented additional contributors to suboptimal breastfeeding, including perceived milk insufficiency, high maternal workload, limited partner involvement, economic constraints such as inflation, and food insecurity (17). The timing and exclusivity of breastfeeding are further influenced by cultural norms, religious beliefs, and socioeconomic factors. Notably, mothers in earlier studies reported insufficient counseling during the perinatal period (10), suggesting gaps in clinical support.

Cesarean delivery emerged as a significant barrier to early breastfeeding initiation. In this study, 80% of mothers were unable to breastfeed within the first hour postpartum—a rate consistent with existing evidence showing lower rates of timely breastfeeding among women undergoing cesarean sections (18). A Canadian study found that 80% of mothers following cesarean delivery encountered breastfeeding difficulties and failed to initiate feeding within the first hour (19). Other studies confirm that cesarean birth is associated with delayed onset or reduced likelihood of breastfeeding (20, 21).

The American College of Obstetricians and Gynecologists identifies improper latching techniques, maternal perception of inadequate milk supply, and premature introduction of complementary foods as leading causes of interrupted exclusive breastfeeding. The World Health Organization recommends initiating breastfeeding within the first hour after birth (22). In our cohort, 81.67% of mothers did not engage in immediate skin-to-skin contact and did not exclusively breastfeed their infants initially. This finding resonates with international data: a UK-based study reported that while 71% of mothers initiated breastfeeding on the first day, few did so within the first hour (23); a Brazilian survey showed that 64% of newborns were separated from their mothers and transferred to nurseries, with 76.8% of these births occurring via cesarean section (19).

Family support has been recognized as crucial for augmenting milk production. In terms of social support, 98% of the mothers in this study indicated satisfaction with family support, while merely 2% reported dissatisfaction. The extant literature corroborates this finding, revealing that supportive environments cultivate maternal confidence, alleviate anxiety, and enhance breastfeeding duration and exclusivity—factors that jointly promote lactogenesis (24). This high level of perceived support parallels previous findings indicating that 95% of mothers felt adequately supported by both family members and healthcare providers during breastfeeding initiation (25). Additional evidence confirms that mothers who receive encouragement from partners, relatives, and hospital staff are more likely to initiate and sustain breastfeeding (26, 27).

In terms of feeding practices, 65% of mothers in this study practiced exclusive breastfeeding, 13.33% used mixed feeding (combining breastfeeding and formula), and 21.67% relied solely on bottle-feeding. Comparable U.S. data indicate that 56 out of 70 mothers breastfed, while 14 adopted mixed feeding—often due to medical complications such as cesarean delivery or perceived low milk supply (28). Furthermore, many mothers in our sample expressed positive emotions toward motherhood and breastfeeding, a sentiment echoed in a Japanese study where 95% of participants reported satisfaction and happiness with both roles (19).

Interestingly, nearly half of respondents (48.63%) denied experiencing milk insufficiency yet still failed to initiate breastfeeding within the first hour post-delivery. This delay may reflect systemic issues rather than individual choice. For context, a Ugandan study found that 26% of mothers perceived insufficient milk production (29) and notably, healthcare providers also commonly report maternal concerns about low supply (29), underscoring its prevalence across settings.

Maternal diet plays a crucial role in lactation success. Nutritional quality directly influences both the composition and volume of breast milk (30). In this study, common dietary components during lactation included chicken, yakhni (a traditional broth), vegetables, and fruits—all potentially beneficial for milk production due to their nutrient density. However, almost half of the mothers did not consume additional meals despite increased energy demands. According to established guidelines, an extra 500 kcal/day is recommended during lactation to meet metabolic needs (30).

This study has several limitations. Data collection from mothers recovering from cesarean sections may have been affected by postoperative discomfort, potentially influencing response accuracy. The use of purposive sampling limits the generalizability of findings to broader populations. Additionally, dietary intake was assessed without standardized tools such as food frequency questionnaires (FFQ) or 24-h dietary recalls, which may affect recall validity. Despite these constraints, to the best of our knowledge, this is the first qualitative study conducted in Khyber Pakhtunkhwa, Pakistan, to systematically explore barriers related to perceived breast milk insufficiency.

## Conclusion

To translate these findings into practice, we propose a dual approach targeting clinical and public health sectors. Clinically, interventions must address the three core barriers identified—delayed breastfeeding initiation, technical knowledge gaps, and cesarean-related challenges—through improved education, standardized support, and monitoring.

At the public health level, policymakers should prioritize creating supportive environments via workplace accommodations, culturally sensitive nutrition campaigns, and community peer networks. This comprehensive strategy is vital for reducing disparities in breastfeeding practices and contributing to the attainment of global nutrition targets.

## Data Availability

The original contributions presented in the study are included in the article/supplementary material, further inquiries can be directed to the corresponding authors.

## References

[1] WHO. Breastfeeding. Available from: https://www.who.int/health-topics/breastfeeding

[2] Gupta S Pajai S Pawade AA. Benefits of breastfeeding on child and postpartum psychological health of the mother. JOGNN. (2023) 15:226–30. doi: 10.5005/jp-journals-10006-2217

[3] Mohebi S Parham M Sharifirad G Gharlipour Z Mohammadbeigi A Rajati F. Relationship between perceived social support and self-care behavior in type 2 diabetics: a cross-sectional study. J Educ Health Promote. (2018) 7:48. doi: 10.4103/jehp.jehp_73_17 29693029 PMC5903155

[4] Pérez-Escamilla R Tomori C Hernández-Cordero S Baker P Barros AJD Bégin F . Breastfeeding: crucially important, but increasingly challenged in a market-driven world. Lancet. (2023) 401:472–85. doi: 10.1016/S0140-6736(22)01932-8 36764313

[5] Babic A Sasamoto N Rosner BA Tworoger SS Jordan SJ Risch HA . Association between breastfeeding and ovarian cancer risk. JAMA Oncol. (2020) 6:e200421. doi: 10.1001/jamaoncol.2020.0421 32239218 PMC7118668

[6] Riaz A Bhamani S Ahmed S Umrani F Jakhro S Qureshi AK . Barriers and facilitators to exclusive breastfeeding in rural Pakistan: a qualitative exploratory study. Int Breastfeed. 17. doi: 10.1186/s13006-022-00495-4 35986337 PMC9389710

[7] Huang Y Liu Y Yu XY Zeng TY. The rates and factors of perceived insufficient milk supply: a systematic review. Matern Child Nutr. (2022) 18:e13255. doi: 10.1111/mcn.13255 34382733 PMC8710095

[8] Ahmed F Malik NI Shahzad M Ahmad M Shahid M Feng XL . Determinants of infant young child feeding among mothers of malnourished children in south Punjab, Pakistan: a qualitative study. Front Public Health. (2022) 10:834089. doi: 10.3389/fpubh.2022.834089 35664102 PMC9160796

[9] Stordal B. Breastfeeding reduces the risk of breast cancer: a call for action in high-income countries with low rates of breastfeeding. Cancer Med. (2023) 12:4616–25. doi: 10.1002/cam4.5288 36164270 PMC9972148

[10] Muhani N Wulandari R Arayastuti N Yanti DE Hermawan D Sefililaisya SN . The relationship between maternal psychology, family, and culture with perception of breast milk insufficiency in breastfeeding mothers of sumur batu public health center, Lampung Indonesia. Malaysian. MJPHM. 20:67–78. doi: 10.37268/mjphm/vol.20/no.3/art.401

[11] Vandenplas Y Basrowi RW. Breastfeeding by working mothers: global challenges. IJCOM. 3:1–2 doi: 10.53773/ijcom.v3i1.82.1-2

[12] Kaß A Dörsam AF Weiß M Zipfel S Giel KE. The impact of maternal eating disorders on breastfeeding practices: a systematic review. Arch Womens Ment Health. (2021) 24:693–708. doi: 10.1007/s00737-021-01103-w 33830375 PMC8492580

[13] Mahurin-Smith J. Challenges with breastfeeding: pain, nipple trauma, and perceived insufficient milk supply. MCN. Am J Matern Child Nurs. (2023) 48:161–7. doi: 10.1097/NMC.0000000000000909 37101329

[14] Song JT Kinshella MLW Kawaza K Goldfarb DM. Neonatal intensive care unit interventions to improve breastfeeding rates at discharge among preterm and low birth weight infants: a systematic review and meta-analysis. Breastfeed Med. (2023) 18:97–106. doi: 10.1089/bfm.2022.0151 36595356

[15] Khan SA Ali N Maroof F Hussain SNF. Barriers to exclusive breastfeeding in mothers belonging to low, middle, and high socio-economic families in Pakistan. Int J Child Health Nutr. 12:32–8. doi: 10.6000/1929-4247.2023.12.01.4

[16] Gizaw AT Sopory P Sudhakar M. Barriers and coping responses towards infant and young child feeding practices in rural Ethiopia: a descriptive qualitative study. BMJ Open. (2023) 13:e077008. doi: 10.1136/bmjopen-2023-077008 37821129 PMC10582866

[17] Does cesarean section have an impact on the successful initiation of breastfeeding in Saudi Arabia? – PubMed. Available from: https://pubmed.ncbi.nlm.nih.gov/25399221/PMC4362139 25399221

[18] Singh J Scime N V Chaput KH. Association of Caesarean delivery and breastfeeding difficulties during the delivery hospitalization: a community-based cohort of women and full-term infants in Alberta, Canada. Can J Public Health. (2023) 114:104–12. doi: 10.17269/s41997-022-00666-0 35902540 PMC9849537

[19] Hobbs AJ Mannion CA McDonald SW Brockway M Tough SC. The impact of caesarean section on breastfeeding initiation, duration and difficulties in the first four months postpartum. BMC Pregnancy Childbirth 16. doi: 10.1186/s12884-016-0876-1 27118118 PMC4847344

[20] Gedefaw G Goedert MH Abebe E Demis A. Effect of cesarean section on initiation of breast feeding: findings from 2016 Ethiopian demographic and health survey. PLoS ONE. (2020) 15:e0244229. doi: 10.1371/journal.pone.0244229 33338080 PMC7748140

[21] Alves R de V de Oliveira MIC Domingues RMSM Pereira APE Leal M. do C. Breastfeeding in the first hour of life in Brazilian private hospitals participating in a quality-of-care improvement project. Reprod Health. (2022) 20:1–11. doi: 10.1186/s12978-022-01538-z 36609292 PMC9817241

[22] Edmond KM Zandoh C Quigley MA Amenga-Etego S Owusu-Agyei S Kirkwood BR. Delayed breastfeeding initiation increases risk of neonatal mortality. Pediatrics. 117. doi: 10.1542/peds.2005-1496 16510618

[23] Priscilla V Afiyanti Y Juliastuti D. A qualitative systematic review of family support for a successful breastfeeding experience among adolescent mothers. Open Access Maced J Med Sci. (2021) 9:775–83. doi: 10.3889/oamjms.2021.7431

[24] Wallenborn JT Wheeler DC Lu J Perera RA Masho SW. Importance of familial opinions on breastfeeding practices: differences between father, mother, and mother-in-law. Breastfeed Med. (2019) 14:560–7. doi: 10.1089/bfm.2019.0049 31298574

[25] Arora S McJunkin C Wehrer J Kuhn P. Major factors influencing breastfeeding rates: mother's perception of father's attitude and milk supply. Pediatrics. (2000) 106:E67. doi: 10.1542/peds.106.5.e67 11061804

[26] Freed GL Fraley JK Schanler RJ. Attitudes of expectant fathers regarding breast-feeding. Pediatrics. (1992) 90:224–7. doi: 10.1542/peds.90.2.224 1641286

[27] Chezem JC. Breastfeeding attitudes among couples planning exclusive breastfeeding or mixed feeding. Breastfeed Med. (2012) 7:155–62. doi: 10.1089/bfm.2011.0024 22224507

[28] Namyalo H Nankumbi J Ngabirano TD. Perceived breast milk insufficiency: prevalence and associated factors among women attending a young child clinic in Uganda. Int J Afr Nurs Sci. (2023) 19:100637. doi: 10.1016/j.ijans.2023.100637

[29] Segura SA Ansótegui JA Marta Díaz-Gómez N. The importance of maternal nutrition during breastfeeding: do breastfeeding mothers need nutritional supplements? An Pediatr. (2016) 84:347.e1–e7. doi: 10.1016/j.anpede.2015.07.035 26383056

[30] Wilson PR Pugh LC. Promoting nutrition in breastfeeding women. JOGNN. (2005) 34:120–4. doi: 10.1177/0884217504272806 15673655

