# Supplementary material for: Mother's perception regarding insufficiency of breast-milk: a qualitative investigation in Haripur, Pakistan
Source: Front Public Health. 2026 Mar 24;14:1742704. doi: 10.3389/fpubh.2026.1742704 (PMC13055618; doi:10.3389/fpubh.2026.1742704)
Supplement: Supplementary file 1 [file Table_1.docx]

**Supplementary file**

The table S1 shows general information about 60 study respondents, who were mothers with ages ranging from 18 to 40 years with children aged 1 day to 6 months. Most respondents were from Haripur, while a few were from other places like Lahore, Islamabad, Abbottabad, Rawalpindi, Bahawalpur, Skardu, and Darvesh. The place of delivery for most respondents was either Haripur or DHQ (District Headquarters Hospital) Haripur.

**S1** **General information of the study respondents**

| **Codes** | **Age of Mothers** | **Age of Childrens** | **Educational Status** | **Occupations** | **Number of Childrens** | **Delivery Places** | **Districts** |
| --- | --- | --- | --- | --- | --- | --- | --- |
| R1 | 24 years | 2 months | Secondary | Housewife | 2 | Haripur | Haripur |
| R2 | 35 Years | 2 months | Tertiary | Housewife | 2 | Lahore | Haripur |
| R3 | 28 Years | 6 Months | Tertiary | Housewife | 4 | DHQ Haripur | Haripur |
| R4 | 27 Years | 20 days | Primary | Housewife | 3 | DHQ Haripur | Haripur |
| R5 | 21 Years | 5 Months | Primary | Housewife | 1 | Haripur | Haripur |
| R6 | 22 Years | 1 day | Primary | Housewife | 1 | Haripur | Haripur |
| R7 | 22 Years | 4 Months | Tertiary | Housewife | 1 | Islamabad | Haripur |
| R8 | 26 Years | 5 Months | Primary | Housewife | 1 | DHQ Haripur | Haripur |
| R9 | 37 Years | 1.2 Months | Primary | Housewife | 10 | DHQ Haripur | Haripur |
| R10 | 37 Years | 1 day | Tertiary | Housewife | 3 | DHQ Haripur | Haripur |
| R11 | 18 Years | 2 days | Primary | Housewife | 2 | DHQ Haripur | Haripur |
| R12 | 38 Years | 3 Months | No | Housewife | 1 | Haripur | Haripur |
| R13 | 25 Years | 3 Months | No | Housewife | 3 | Haripur | Haripur |
| R14 | 30 Years | 1 day | Primary | Housewife | 3 | DHQ Haripur | Haripur |
| R15 | 30 Years | 4 Months | No | Housewife | 5 | Civil Hospital | Haripur |
| R16 | 34 Years | 4 Months | Primary | Housewife | 3 | Abbottabad | Haripur |
| R17 | 32 Years | 3.5 Months | Primary | Housewife | 5 | Haripur | Haripur |
| R18 | 23 Years | 1 Months | Tertiary | Housewife | 2 | DHQ Haripur | Haripur |
| R19 | 30 Years | 2 days | Tertiary | Housewife | 3 | DHQ Haripur | Haripur |
| R20 | 32 Years | 6 Months | No | Housewife | 5 | Haripur | Haripur |
| R21 | 32 Years | 2 days | Tertiary | Housewife | 3 | DHQ Haripur | Haripur |
| R22 | 19 Years | 1 day | Secondary | Housewife | 1 | DHQ Haripur | Haripur |
| R23 | 21 Years | 3.5 Months | Tertiary | Housewife | 2 | Lahore | Lahore |
| R24 | 24 Years | 2 days | Secondary | Housewife | 2 | DHQ Haripur | Haripur |
| R25 | 26 Years | 1 Months | Primary | Housewife | 1 | Haripur | Haripur |
| R26 | 21 Years | 1 Months | No | Housewife | 4 | Darvesh Haripur | Haripur |
| R27 | 30 Years | 2 Months | No | Housewife | 4 | Haripur | Haripur |
| R28 | 27 Years | 1.2 Months | Tertiary | Housewife | 1 | DHQ Haripur | Haripur |
| R29 | 18 Years | 1.5 Months | Primary | Housewife | 1 | DHQ Haripur | Haripur |
| R30 | 24 Years | 1.5 Months | Primary | Housewife | 1 | DHQ Haripur | Haripur |
| R31 | 24 Years | 2 Months | No | Housewife | 2 | Haripur | Haripur |
| R32 | 26 Years | 12 days | Primary | Housewife | 4 | Darvesh Haripur | Haripur |
| R33 | 19 Years | 1 Months | Primary | Housewife | 1 | Haripur | Haripur |
| R34 | 27 Years | 3 days | Primary | Housewife | 2 | DHQ Haripur | Haripur |
| R35 | 35 Years | 15 days | Tertiary | Housewife | 3 | Haripur | Haripur |
| R36 | 25 Years | 1 day | No | Housewife | 3 | DHQ Haripur | Haripur |
| R37 | 40 Years | 1 day | No | Housewife | 3 | DHQ Haripur | Haripur |
| R38 | 32 Years | 4 Months | No | Housewife | 6 | DHQ Haripur | Haripur |
| R39 | 30 Years | 2 days | No | Housewife | 4 | Haripur | Haripur |
| R40 | 18 Years | 22 days | No | Housewife | 1 | DHQ Haripur | Haripur |
| R41 | 20 Years | 1 day | Secondary | Housewife | 1 | DHQ Haripur | Haripur |
| R42 | 28 Years | 3.5 Months | Tertiary | Housewife | 2 | Rawalpindi | Haripur |
| R43 | 21 Years | 6 Months | Primary | Housewife | 1 | DHQ Haripur | Haripur |
| R44 | 22 Years | 6 Months | No | Housewife | 3 | Abbottabad | Haripur |
| R45 | 23 Years | 3.5 Months | Primary | Housewife | 3 | Bahawalpur | Punjab |
| R46 | 35 Years | 2 Months | Secondary | Housewife | 3 | DHQ Haripur | Haripur |
| R47 | 25 Years | 5 Months | Tertiary | Housewife | 2 | Skardu | Gilgit |
| R48 | 30 Years | 1 Months | Tertiary | Housewife | 4 | Haripur | Haripur |
| R49 | 34 Years | 3 days | Secondary | Housewife | 3 | DHQ Haripur | Haripur |
| R50 | 28 Years | 3 Months | No | Housewife | 2 | DHQ Haripur | Haripur |
| R51 | 20 Years | 15 days | No | Housewife | 1 | DHQ Haripur | Haripur |
| R52 | 30 Years | 1 day | Primary | Housewife | 4 | Haripur | Haripur |
| R53 | 30 Years | 3 Months | Tertiary | Housewife | 1 | Yahya Hospital | Haripur |
| R54 | 20 Years | 11 days | Primary | Housewife | 1 | Yahya Hospital | Haripur |
| R55 | 26 Years | 1 day | Primary | Housewife | 3 | Haripur | Haripur |
| R56 | 30 Years | 2.5 Months | Secondary | Housewife | 1 | Haripur | Haripur |
| R57 | 25 Years | 2 days | Primary | Housewife | 2 | Haripur | Haripur |
| R58 | 24 Years | 4 Months | Primary | Housewife | 1 | Haripur | Haripur |
| R59 | 28 Years | 3 Months | Tertiary | Housewife | 1 | Abbottabad | Haripur |
| R60 | 30 Years | 2 days | No | Housewife | 2 | DHQ Haripur | Haripur |
